# What is community engagement and how can it drive malaria elimination? Case studies and stakeholder interviews

**DOI:** 10.1186/s12936-019-2878-8

**Published:** 2019-07-17

**Authors:** Kimberly Baltzell, Kelly Harvard, Marguerite Hanley, Roly Gosling, Ingrid Chen

**Affiliations:** 1 Department of Family Health Care Nursing, University of California San Francisco School of Nursing, San Francisco, USA; 2 Institute for Global Health Sciences, University of California San Francisco, San Francisco, USA; 3 Malaria Elimination Initiative, Global Health Group, University of California San Francisco, San Francisco, USA; 4 Multidisciplinary Research Centre, University of Namibia, Windhoek, Namibia

**Keywords:** Community engagement, Malaria elimination, Community participation, Local leadership, Community buy-in, Community implementation

## Abstract

**Background:**

In light of increasing complexity of identifying and treating malaria cases in low transmission settings, operational solutions are needed to increase effective delivery of interventions. Community engagement (CE) is at the forefront of this conversation given the shift toward creating local and site-specific solutions. Malaria programmes often confuse CE with providing information to the community or implementing community-based interventions. This study seeks to expand on CE approaches for malaria by looking to a variety of health and development programmes for lessons that can be applied to malaria elimination.

**Methods:**

Qualitative data was collected from key informant interviews and community-based focus group discussions. Manual analysis was conducted with a focus on key principles, programme successes and challenges, the operational framework, and any applicable results.

**Results:**

Ten programmes were included in the analysis: Ebola, HIV/Hepatitis C, Guinea worm, malaria, nutrition, and water, sanitation and hygiene. Seven focus group discussions (FGDs) with 69 participants, 49 key informant (KI) interviews with programme staff, and 7 KI interviews with thought leaders were conducted between October–April 2018. Participants discussed the critical role that village leaders and community health workers play in CE. Many programmes stated understanding community priorities is key for CE and that CE should be proactive and iterative. A major theme was prioritizing bi-directional interpersonal communication led by local community health workers. Programmes reported that measuring CE is difficult, particularly since CE is ongoing and fluid.

**Conclusions:**

Results overwhelmingly suggest that CE must be an iterative process that relies on early involvement, frequent feedback and active community participation to be successful. Empowering districts and communities in planning and executing community-based interventions is necessary. Communities affected by the disease will ultimately achieve malaria elimination. For this to happen, the community itself must define, believe in, and commit to strategies to interrupt transmission.

**Electronic supplementary material:**

The online version of this article (10.1186/s12936-019-2878-8) contains supplementary material, which is available to authorized users.

## Background

Between 2000 and 2015, 17 countries successfully eliminated malaria and an additional 10 countries are expected to eliminate by 2020 [1]. Based on this success, malaria eradication is being explored as a feasible global goal. Despite initial momentum, however, progress has stalled and actually regressed in some countries, primarily in the WHO African Region [2]. Funding for malaria control and elimination has remained relatively stable over the past 2 years, as have reported cases at approximately 219 million cases of malaria in 2017 [2]. In countries that have successfully decreased their malaria burden, cases often start to cluster in smaller geographic foci and among subpopulations with unique risk characteristics [3, 4]. Current interventions, including insecticide-treated bed nets, indoor residual spraying (IRS), and community case management are effective only if they are accessible, acceptable, and properly used within communities. Many of the challenges to malaria elimination are site specific and require a more tailored approach to effectively target these remaining foci of transmission and populations at higher risk.

In light of stalled progress, flatlined funding [2], and the increasing complexity of identifying and treating malaria cases, the global community is looking to operational solutions to increase the effective delivery of interventions for malaria. The topic of community engagement is at the forefront of this conversation and is recognized as an essential component in the shift toward creating local and site-specific solutions. Community engagement strategies have long been incorporated into themes such as women’s health, political action, and HIV [5]. Community engagement is often confused with simply providing information, education, and communication (IEC) to the community; the malaria community have only recently begun to consider the significance and potential of community engagement for malaria elimination [6–9].

Achieving effective community engagement in malaria will be a major challenge since most elimination settings must grapple with heterogeneous transmission, often concentrated in hard to access locations and/or among marginalized population groups [3], as well as changes in perceptions of personal risk and community-level health priorities [7]. Malaria control and elimination programmes often employ standard community-based activities for malaria including the use of community health workers or volunteers (CHWs) to conduct case management, surveillance, vector control, and information, education and communication (IEC) activities and social behaviour change communication (SBCC). This study seeks to expand on potential community engagement approaches for malaria by looking to lessons from a variety of health and development programmes.

There is great potential to improve community engagement for malaria elimination. It is possible that other health and development sectors can help to inform a paradigm shift in how malaria programmes incorporate affected communities in interventions. This series of case studies aims to capture the experience of community engagement across a range of health and development sectors to explore how malaria and other programmes use community engagement strategies to design, implement, monitor, and sustain interventions.

## Methods

### Overview

This qualitative study used key informant (KI) interviews and focus group discussions (FGDs) to explore approaches to community engagement in malaria elimination efforts as well as other sectors. Programmes from eight health and development sectors, including malaria, were identified for programmatic evaluation. Specifically, the study explores community engagement perceptions and practices at three levels; from thought leaders (defined as those with expertise or leadership positions in sectors included in the study) who design CE activities, from programmatic staff who manage and implement community engagement activities, and from community members involved in community engagement interventions.

Participants were identified from within the researchers’ network and/or snowball sampling. Individuals were contacted through email to ask if they might be a suitable participant in the study; could provide contact information for other individuals with relevant contacts; and could provide contact information for other potential participants. Through this process, relevant and accessible individuals and institutions with current or prior experience working on community engagement programs were identified.

### Programme inclusion/exclusion criteria

Programmes selected for inclusion met the following criteria: (1) from priority health and development sectors identified by the research team; (2) represent a varied selection of sectors and institution-types; (3) contain an intentionally designed community engagement strategy; (4) perceived by colleagues and/or programme staff as successfully mobilizing community action, and/or having taken a creative, bottom-up approach to engaging the community; (5) from geographically diverse locations; and (6) KIs representing the programme were able to be identified through the research team networks. Programmes that did not address health or development issues and those which did not employ community engagement activities were excluded from consideration.

### Thought leaders

Thought leaders were interviewed using a semi-structured interview guide (Additional file 1) designed to explore the role that community engagement has played across different health and development sectors, and to establish similarities and differences in approaches to community engagement practices. The term “thought leader” is described as those who possess social capital, have respect and influence among colleagues, and can influence others [10].

Inclusion criteria for thought leaders included in this study are: (1) ≥ 5 years in a senior-level position within the health and development sector; (2) previously involved or currently involved in community engagement strategy and design; (3) ≥ 18 years of age; (4) fluency in English; and (5) willing to provide written informed consent. Interviews were conducted in-person when feasible, and by Skype or phone when travel by the research team was not feasible. When available, a note taker was present, and interviews were audio recorded when permission from the key informant was granted. Written consent from the interviewee was obtained prior to the start of the interview and interviews took no longer than 60 min. Individuals were excluded if they are unable or unwilling to provide informed consent.

### Programme staff

For each programme, between two and eight programme staff were interviewed following a semi-structured interview guide. Samples questions focused on programme objectives, measurements of success, sources of guidance, and both explicit and perceived definitions of community engagement (Table 1). Interviews focused on the community engagement strategies employed including the design process; operational, financial and human resource requirements; key elements; lessons learned and any available results; as well as the contextual factors that may have positively or negatively impacted the programme.

**Table 1:** Constructs and sample questions from key informant interview guides

Construct	Sample question
General programme objectives	What are the programme’s stated objectives? Were there any informal goals of the programme that were not explicitly included?
Measurement of success	Was there a way to measure the success or failure of the community engagement strategies used in your programme? If yes, what were they?
Sources of guidance	Are there any important information sources on general community engagement that you would recommend?
Meaning of community engagement	What does community engagement mean to you?

Programme staff selected for key informant interviews met the following criteria: (1) ≥ 6 months organizational experience with the ability to discuss at length the selected community engagement programme including the design process, key elements, operational framework, financial and human resource requirements, and/or results; (2) involved in the community engagement strategy design, implementation and/or assessment; (3) employed in the organization of the selected community engagement effort within the past 3 years and has organizational permission to discussion the programme; (4) ≥ 18 years of age; (5) fluency in English; and (6) willing to provide written informed consent. Paid community health workers were also considered programme staff for purposes of this study and these interviews were conducted with the corresponding interview guide.

### Community members

In collaboration with the participating programmes, FGDs with individuals residing in the programme catchment areas were conducted. Participating programme staff and community leaders assisted in the identification of focus group participants; purposive sampling and/or snowball sampling was used when necessary. Focus group participants were identified based on their place of residence during programme implementation, as well as their willingness to participate. An attempt was made to ensure that both men and women were included in each FGD.

Focus group discussions sought to obtain the community’s perception of programme activities and outcomes to examine motivators and impediments to community engagement. A semi-structured interview guide was used to frame the discussions and all FGDs were conducted in-person during field visits by a member of the research team. Questions focused on the meaning of community engagement, impressions of past experience with community engagement strategies, and how future efforts could be improved (Table 2).

**Table 2:** Constructs and sample questions from community focus group discussions

Construct	Sample question
Personal experience with community engagement	How did you learn about the community engagement project? Was the community involved in helping with the project before it started? During? After?
Suggestions for the future	If you were designing a community project what activities would you implement and how would you involve different groups of people?

Participants selected for FGDs met the following criteria: (1) familiarity with the selected community engagement programme, (2) resided in the programme catchment area at the time of implementation of the community engagement strategies, (3) ≥ 18 years of age, and (4) willing to provide written informed consent. Unpaid community health workers were considered community members for the purposes of this study. When English was not the native language of participants, a local translator was used. Focus group discussions took no longer than 90 min. Light refreshments were offered to FGD participants. Individuals were excluded if they were unable or unwilling to provide informed consent.

### Data analysis

After each interview or FGD, the interviewer recorded themes, general comments, and additional observations. Audio-recordings were not transcribed word-for-word. Instead, after each interview or FGD, detailed notes were recorded, and a discussion of themes and observations occurred between the interviewer and note-taker when a note-taker was present. After the interview or FGD, notes were uploaded into Microsoft Word (2010). The data were manually analysed to obtain a greater depth of knowledge on the health and development programmes profiled, with a focus on key principles, the programme successes and challenges, lessons learned, the operational framework, and any applicable results.

## Results

Overall, ten programmes with community engagement strategies from seven different health focus areas were included in the analysis: Ebola, HIV/Hepatitis C, Guinea worm, malaria, nutrition, and water, sanitation, and hygiene (WASH) (Table 3). Seven focus group discussions (FGDs) with 69 participants, 49 key informant interviews with programme staff, and seven key informant interviews with thought leaders were conducted between October 2017 and April 2018. Four of the ten programmes focus on disease elimination and eradication (The Carter Center, Isdell:Flowers, PATH MACEPA, and MORU).

**Table 3:** Programmes included in analysis and level of engagement

Program name	Location	Health focus area	Type of institution	# Program staff interviewed	# of FGDs conducted and # of participants	# Thought leaders interviewed
The Carter Center	Angola, Chad, Ethiopia, Mali, South Sudan	Guinea worm	NGO	2	–	1
GAIA	Malawi	HIV	NGO	17	1 (6 participants)	n/a
Institute for Global Health Sciences, UCSF	Global	Global Health	Academia	–	–	1
Isdell:Flowers	Angola, Namibia, Zambia, Zimbabwe	Malaria	NGO	3	1 (10 participants)	–
Kore Timoun	Haiti	Nutrition	NGO	4	2 (25 participants)	–
PATH MACEPA	Ethiopia, Kenya, Senegal, Zambia	Malaria	NGO	–	–	1
MORU	Asia Pacific (+Democratic Republic of Congo)	Malaria and NTDs	Research Institute	–	–	1
Belize red cross	Belize	WASH	NGO	4	2 (14 participants)	–
Treat Asia/amfAR	Thailand	HIV and Hepatitis C	NGO and foundation	8	–	1
Wellbody Alliance	Sierra Leone	Ebola virus disease	NGO	11	1 (14 participants)	2

*FGDs* focus group discussions, *GAIA* Global AIDS Interfaith Alliance, *HIV* human immunodeficiency virus, *MORU* the Mahidol Oxford Tropical Medicine Research Unit, *NGO* non-governmental organization, *NTDs* neglected tropical diseases, *PATH MACEPA* PATH’s Malaria Control and Elimination Partnership in Africa, *TREAT Asia/amfAR* Therapeutics Research, Education, and AIDS Training in Asia/American Foundation for AIDS Research

### Key stakeholders to include in the design and implementation of community engagement process

Programmes included in this study reported targeting and working with a range of stakeholders on community engagement including: local and religious leaders, nurses and community health workers (CHWs), village councils and other community groups (including women’s groups, youth groups) and teachers and school children. Many of the KI interviews and FGDs centered on the critical role that village leaders and CHWs play in community engagement efforts.

Consultation with and buy-in from community leaders such as village chiefs, pastors, and local healers was noted to be critical by all ten programmes. Several programmes referred to community leaders as “key influencers,” and emphasized that programmes are likely to fail if local leadership does not support the programme’s goals. *“[Village leaders are the] gatekeepers in Malawi*—*you must work with them to understand the cultural factors at play. Their input is invaluable*.” (KI participant). It was reported that collaborating with village leaders could often help identify other potential community leaders; particularly CHWs, which many programmes rely on to deliver community engagement and other health services. All programmes that recruited CHWs from the community used local leadership to identify appropriate candidates.

Many of the programmes support the practice of hiring known and trusted community members as CHWS. Programmes also reported leveraging nurses, teachers, community health workers, and targeted peer groups to reach these groups extended networks. Peers include women and mothers, and at least one programme established a men’s group after finding that there was no formal mechanism to engage with this population at the community level. In addition, several groups reported success in developing survivors and/or people living with the disease of focus into CHWs or programme staff/volunteers for greater impact and credibility among those with similar prognoses (e.g. people living with HIV, TB, Ebola survivors).

Working with the Ministry of Health (MoH) was cited as critical by most programmes; however, it was also noted that MoH priorities can change and there are often competing demands for focus. Several programmes pointed to the importance of partnering with district health authorities. Doing work beyond the primary health focus (e.g. providing school fees and/or uniforms for orphans, providing family planning or child vaccination services) was described by several programmes, indicating the value of a multi-sectoral approach to community engagement.

### Ensuring buy-in

Many of the programmes in this project use creative and socially-based approaches to advance engagement. Utilizing multiple channels of communication were cited as important. Examples of channels used by the programmes in this study include the use of soccer clubs, films, community drama, television, songs, Facebook, social events such as weddings, discos, and community fairs with prizes. Children were repeatedly mentioned as important change agents and it was recommended that youth groups be mobilized to assist with messaging: “*kids are the channel to the homes*.” (KI participant). One programme was having difficulty obtaining community buy-in so they expanded outreach into schools where they were able to successfully engage parents by sharing programme goals and messaging with students.

As a mechanism for representing collective stakeholder interests and showing neutrality, Belize Red Cross brought together groups of local residents (30–40 people) to do mapping exercises around water sources. Activities that involve everyone and do not have a political or religious agenda were deemed important in this setting; however, using religious leaders for messaging was cited as valuable in other contexts. Several programmes reported that providing malaria messaging at church was a particularly useful strategy and one KI cited the power of having the local pastor take his anti-malarial medicine during his sermon. In areas where mobile technology is readily available, it was used by some programmes to engage the community; this included using apps to report cases or disseminate information, education and communication (IEC) materials.

One thought leader cautioned that community priorities may not be the same as the programme priorities and three programmes recommended openly identifying and acknowledging competing priorities in discussions with community stakeholders. Kore Timoun asks communities to apply for help with their locally identified needs and if they align with Kore Timoun’s mission and capacity, a partnership is formed. Four programmes reported that incorporating community-identified or acute health priorities into the primary programme was found to be useful. In fact, several of the organizations profiled here started with a specific health or development focus and expanded to other content areas after infrastructure was in place and the community was familiar with the work of the organization. A striking example is Wellbody Alliance, which initially started with the goal of providing primary care. In order to deliver high-quality primary care, Wellbody ran community-based HIV, TB, maternal, and child health programmes. During the 2014–2016 Ebola outbreak in Sierra Leone, the existing community health worker infrastructure and organization’s approach to community engagement was leveraged to rapidly develop community-based Ebola virus disease surveillance programmes.

One of Wellbody Alliance’s major learnings from the Ebola outbreak was the power of bi-directional, interpersonal communication led by local CHWs, and other programmes profiled in the study echoed this. Community meetings are popular venues for community engagement and were cited by six of the programmes. Other popular group activities include drama and music performances, art shows, and school-based activities. Door-to-door engagement with the local community was cited as being very effective by programmes using the technique; one programme reported targeting home visits to community members that often miss larger events or to individuals who seem hesitant or unwilling to participate. In these instances, discussions center on specific concerns, perceptions, or challenges to participation. Similarly, to engage with more people, the majority of programmes suggested that the timing of programme activities should accommodate various work and holiday schedules. Several programmes noted that when interventions or education sessions are only offered Monday through Friday during the day, community members who work or travel are often missed.

Among the programmes included in this study, there was variation in approaches to increasing community motivation. Several programmes use reward systems and several use community members to identify those who do not participate or are chronically missed by existing interventions. One programme provides cash rewards to those who reported a suspected case of disease (The Carter Center), while others use prizes to recognize community members (GAIA). Another programme (Belize Red Cross) created a cleanup campaign, which received formal recognition from the local Department of Environment for providing the community with the most improvement in hygiene; programme staff and community members stated that the community remains clean today.

TREAT Asia, an organization focused on HIV and co-infections including Hepatitis C, reported that harnessing the anger at being marginalized based on health status was key to the success of their work. They stated that those infected are highly motivated to advocate for others affected by HIV. To reduce stigma, several programmes use harmonization with programmes targeting other health and development areas; for example, asking “how does HIV affect agriculture?” delivers messaging without segregating a specific group. Three programmes also reported that positive messaging was almost always more effective than scare tactics. Kore Timoun bases its community engagement approach on the positive deviance model, which identifies individuals in the community who face the same socioeconomic and cultural conditions as peers but have established personal practices that keep themselves and/or family members healthy. These individuals are then empowered to share their successful practices with other members of the community [11].

The majority of the programmes included in this report acknowledged that understanding community priorities is key. Most programmes’ goals include moving communities from focusing on treatment to prevention. However, the first step in achieving this shift is to work with local leaders to confirm that the programme priority is among the community’s main concerns. “*Villages have their own priorities, and most people need to see some self*-*interest in taking up additional activities*.” (Thought leader). Regardless of the specific approach or activity, there is general consensus that collaborating with the community is essential, especially as a means to promote community ownership and sustainability of the health or development programme. Treating the community as a partner was a common theme.

### Community engagement needs to be a proactive and iterative process

Seven of the programmes reported that community engagement should be proactive and iterative. Most KIs were in agreement that for community engagement to be effective, it should be integrated from the start and should not only be implemented once a problem has been identified. It was noted that communities should have full and transparent information regarding the programme upfront: “*The community must have detailed information on the programmes intentions, goals, timeline, exit strategy… They deserve full transparency, and when they get it they are usually much more engaged.*” (KI participant). Most programmes discussed taking the programme design to the community after it was finalized. However, they suggested it is important to include the community in the initial design of the intervention or programme and then find ways to continually engage stakeholders throughout the process.

Participants described CE as a “learning process,” and strongly advocated that the community engagement efforts be responsive to the changing perceptions and needs of the community. “*The nature of community engagement is that it needs to be constantly modified*.” (KI participant). Being able to respond to rumors or changing attitudes and perceptions by shifting strategies, activities, and/or targeting and engaging with new or different groups quickly and effectively, was identified as a key factor of successful community engagement programmes. Establishing a transparent feedback loop was also cited as important for community engagement. Similarly, giving community tangible feedback on programme results and milestones was mentioned as vital to keep communities invested. One focus group participant reinforced the importance of continuous communication with the community before, during, and after an intervention. In this programme, providing “complete information” along with consistent messaging, sensitization, and dialogue were key components to keeping the community engaged. This sentiment was echoed by another thought leader who suggested the development of a community engagement task force to lead and facilitate the iterative process.

### Challenges with measurement of success

Programmes included in this analysis have a range of goals, from eliminating disease, to creating cadres of community health workers, improving health literacy, and empowering communities. However, many of the programmes reported that measuring community engagement is difficult. While easy to quantify results from surveys and other tools, most programmes said it was difficult to measure which aspects of community engagement are most effective. This was stated as particularly difficult given that community engagement is generally an ongoing and fluid process with no fixed set of strategies. Most programmes in this study proactively engage with communities and continuously adapt as needed. One programme suggested doing resident surveys to assess community satisfaction and sense of ownership. Another programme used the number of community members seeking HIV testing as a monitoring and evaluation metric—if the number fell below the target, the organization went to the community to find out why and changed engagement strategies once they received feedback.

Some of the strongest evidence of successful community engagement are examples of community-initiated programmes that supported, enhanced, or built upon the original intervention. Similarly, if the community asked for additional services to facilitate participation, this was also seen as a sign of successful community engagement. In the case of GAIA, CHWs continued to do their job even after the programme ended—their feeling of ownership created a sense of responsibility to the community. One programme was contacted by the MoH to train workers, including teachers, in districts outside the original scope of the programme to expand their mission and reach. In one malaria programme, the community independently instituted border screening to prevent constant reintroduction of the disease by a neighbouring country.

Several organizations use traditional metrics to measure success; for example, the number of patient visits to clinics, increased use of bed nets, decrease in number of HIV cases diagnosed, decreased use of traditional healers, reduced number of births due to uptake of family planning, and decrease in number of children diagnosed with malnutrition. In addition, regular stakeholder meetings, satisfaction surveys, and group discussions were listed as ways to gauge the success of community engagement strategies and remind the community that the programme values their input and engagement.

## Discussion

Without exception, all participants in this study agreed that community engagement is vital for long-term success of any intervention or for uptake of new strategies to improve health. This sentiment is echoed in various global technical strategies and resolutions. However, how to operationalize community engagement at scale remains elusive and participants in this study acknowledge that the definition and execution of community engagement varies greatly. The majority of participants included in this study point to some consensus that transformative community engagement is more than providing information to the community, and that communities should be involved in the design and implementation of health interventions. There are some consistent community engagement practices that support a collaborative approach to community engagement and are worth noting by malaria elimination programmes.

First, guidance and support by local leadership before developing a strategy was cited as critical. Community leaders have long been identified as essential to community engagement. Lavery et al. state that it is important to know if the community wants what is offered before setting an agenda [5]. During the 2014–2016 Ebola outbreak in West Africa, establishing strategic partnerships with community and religious leaders was identified as one of seven key domains for success of the response [12].

Second, virtually all programmes included in this study rely on CHWs to be the primary point of contact between the organization and the community. CHWs play an important role in community engagement and their presence was identified as necessary to gain community trust and acceptance. To strengthen community buy-in, programmes using CHWs reported that their CHWs are nominated by local leadership and/or through a participatory, community-based election process. A few programmes reported that for CHWs to be truly effective, they must have a consistent presence in the community. In malaria, the disjointed nature of hiring CHWs for short-term or seasonal intervention work may limit their effectiveness. Understanding how to best train and use CHWs in low transmission settings is important for malaria elimination given that CHWs are often the first point of contact for febrile illness [13]. In Myanmar, expanding the remit of “malaria-only” CHWs to include a broader package of basic health care services has proven effective in sustaining community uptake of malaria services in low transmission areas of the country [14]. Additionally, having established CHWs within the community prior to malaria or other infectious disease outbreaks may prove invaluable during response measures, as it was for Wellbody Alliance during the Ebola epidemic in Sierra Leone. Mobilizing adequate human resources to support the community engagement is important but is only one piece of a larger process [15]. A recent systematic review shows investing in CHWs performance may result in improved behavioural outcomes for patients and an increase in the number of patients seeking care, important aspects of malaria elimination. Practices and interventions noted to improve CHW performance include (1) emphasizing personal career growth; (2) encouraging CHW supervisors to remind CHWs of tasks and hold accountable those who are underperforming; (3) creating proper incentives based on responsibilities of the CHW; and (4) using mobile phone technology when available [16]. While CHWs were identified as enabling community engagement, it is important not to conflate CHW programmes with community engagement.

Third, is the importance of bidirectional communication. Research often focuses on increasing the number of participants in a study, usually through sensitization. This is a unidirectional process, delivering messaging after an intervention has been decided upon. While sensitization has been shown to result in improved uptake during a research study or clinical trial [17], health interventions or strategies that remain in the community long-term may benefit from bidirectional communication. In fact, bidirectional learning was identified as a key component in interventions where population health and/or health behaviour improved [18]. The “community dialogue” approach offers one possible mechanism for strengthening community engagement and uptake of health services [19]. Study results and recent literature indicate that home-based visits to discuss individual and/or household-level concerns related to acceptance and uptake of interventions may be useful [20, 21]. It is relevant to note that all programmes stated that community engagement should be iterative and responsive to changing community needs, perceptions, and opinions. Another likely challenge to malaria elimination activities are community misconceptions and rumors: lessons from polio elimination show the often detrimental power that rumors can exert on disease eradication and elimination programmes [22]. The importance of ongoing efforts to adapt both programming and messaging based on the specific needs of a community further suggests that community engagement is not a one-size-fits-all strategy.

Fourth, is consideration of the possible disconnect between local residents and the ministry officials making decisions on behalf of the community. In addition, while harmonization across health and development sectors is important to avoid community participation burn-out, harmonization within sectors was noted as important as well; malaria interventions should aim to avoid duplicative efforts with other programmes, and engage with local residents in advance of the start of control or elimination activities. As indicated above, the programmes included in this study reported that obtaining community input into the design itself of the health intervention, prior to implementation, is important. This was cited even among programmes that do not actually practice this, possibly indicating that operationalizing such learning is difficult. A community engagement strategy that is iterative, harmonized, and tailored to the local context is difficult to manage at the national level; since malaria elimination strategies are carried out at the district level, it is fitting that local teams should be empowered to develop, adapt, and implement community engagement strategies.

Finally, given the few number of cases in low malaria transmission settings, it is possible that communities no longer identify malaria as the greatest threat to well-being—a serious challenge to these lessons. In Eswatini, younger members of the community were less likely to have had malaria or to have seen the effects of malaria firsthand, limiting their concern of risk or personal investment in elimination efforts (Baltzell, in press). One of the key informants interviewed reported that moving from primarily curative to preventive services merits careful consideration. Studies show that village-based malaria workers are often more effective at providing diagnosis and treatment than prevention and vector control [23]. Additionally, in recent targeted malaria elimination pilot studies in the Greater Mekong Sub-region, the health messaging, particularly on the role of asymptomatic infections, is reported as complex and difficult to communicate effectively [20, 21].

Study participants acknowledged the difficulty of measuring the impact of community engagement. It was often reported that community engagement is time- and resource-intensive. However, instead of traditional outcomes measures, improved community health may be confirmation enough of community engagement and its importance. Methods cited to evaluate the community engagement process include process evaluations, FGDs, and KAP surveys. Rifkin et al. developed an approach to for assessing engagement based on a continuum of participation [24]. This tool is adaptable and provides at least one method to evaluate the process of engagement and highlight its links to health outcomes. This evaluation process can be further strengthened by involving community stakeholders in the collection and analysis of data [25].

One programme reports it is important to critically assess the diversity of those involved in the community engagement process. This is supported by various sets of normative guidance including the Principles of Community Engagement, released by the US Center for Disease Control (CDC) and Agency for Toxic and Substances and Disease Registry (ASTDR), which recommend evaluating the following questions during the M&E process for community engagement [26]:

- Are the right community members at the table?
- Does the process and structure of meetings allow for all voices to be heard and equally valued? For example, where do meetings take place, at what time of day or night, and who leads the meetings?

These are especially important considerations for malaria elimination, as those considered to be the “right” community members will shift and evolve as transmission decreases and malaria increasingly affects different and heterogeneous groups and individuals.

In summary, all programmes in this study prioritized community engagement strategies in their work. Overriding principles among the study participants include listening to the voice of the community and its leaders prior to study/intervention design and not simply offering sensitization during or after implementation; expanding the use and training of community members to help with programme delivery (e.g., CHWs); and making sure that community engagement is an ongoing and iterative process. One key informant described community engagement as an “art”. It might also be considered a programme management issue. To improve implementation, community engagement should be delivered and managed sub-nationally. It should be moved out of isolation within programmes, harmonized with other health programmes, and embedded within local health priorities. When appropriate, malaria elimination programmes should consider closer coordination with other health and development sectors; focusing messaging on prevention versus treatment and the importance of testing in the absence of symptoms; and contributing to the development of a permanent, cohesive team of CHWs and/or a community engagement task force to assist with sustainability of multiple health programmes. In particular, malaria elimination settings may be hindered by a perception of no risk among populations where cases are few. Given this scenario, community engagement will take on added importance to make sure that communities themselves have the capacity and resources to lead sustainable, effective measures to reduce and eventually eliminate malaria transmission.

### Limitations

All successful elements of community engagement reported here are self-reported and the study team did not independently assess impact. While this study was comprehensive in the scope of programmes and stakeholders, purposive sampling was used to identify participants and this may have influenced results. In addition, focus group discussions and KI interviews were not transcribed word for word; the analysis was based on summaries written by the interviewing field team. This limited the authors’ ability to illustrate findings with verbatim quotes or messaging. Lastly, only academic institutions and NGOs were included among the participating programmes. However, despite these limitations, study findings align with much of the literature on the topic of community engagement.

## Conclusions

Evidence from the case studies overwhelmingly suggests that community engagement must be an iterative process that relies on early involvement, frequent feedback, and active participation from the community to be successful. While there are other papers that explore the role of community engagement in malaria elimination, this study offers several conclusions to expand on that exploration.

First, strengthening other complementary health programmes is likely to improve community engagement and community participation, a prime example being improving CHW programs and incorporating community engagement best practices, such as involving the community in the CHW recruitment process. Many of these strategies and policy documents are already endorsed by WHO and its country partners; it is important to begin to prioritize the practical implementation of these strategies and policies more broadly.

Second, much of the necessary infrastructure (i.e. CHW programmes) and processes (i.e. community dialogue and other deliberative processes) exist but likely require quality improvement interventions. The processes and accountability mechanisms for involving communities in decision making should be built into programmes prior to implementation. To make this shift, malaria programmes will need support from specialists in participatory methodology and community engagement, amongst others. The good news is that such expertise exists, as demonstrated in this paper.

Third, is that in order for community engagement to be an iterative process that relies on early involvement, frequent feedback, and active participation from the community, malaria programmes will need to empower districts and communities to become involved in planning and executing community-based interventions. Units closer to the level of implementation should be better equipped and empowered to lead community engagement efforts. This would be a departure from how many national malaria programs operate today. This shift would not only instill the operational nimbleness required for community engagement but would also promote the implementation of locally tailored and targeted case management, vector control, and surveillance interventions.

Malaria elimination will ultimately be achieved by the communities affected by the disease; for this to happen, the community itself must define, believe in, and commit to strategies to interrupt transmission.

## Additional file

**Additional file 1.** Questionnaire and interviewer guide: thought leader interviews from different health and development sectors .

## Data Availability

The datasets/transcript briefs analysed during the current study are available from the corresponding author on reasonable request.

## Abbreviations

ASTDR: Agency for Toxic and Substances and Disease Registry
CDC: Center for Disease Control
CHWs: community health workers or volunteers
FGDs: focus group discussions
IRS: indoor residual spraying
IEC: information, education, and communication
KI: key informant
MoH: Ministry of Health
SBCC: social behaviour change communication
WASH: water, sanitation, and hygiene
