# Supplementary material for: What is community engagement and how can it drive malaria elimination? Case studies and stakeholder interviews
Source: Malar J. 2019 Jul 17;18:245. doi: 10.1186/s12936-019-2878-8 (PMC6637529; doi:10.1186/s12936-019-2878-8)
Supplement: Supplementary file 1 — Additional file 1. Questionnaire and interviewer guide: thought leader interviews from different health and development sectors . [file 12936_2019_2878_MOESM1_ESM.docx]

## Additional file 1: Questionnaire and interviewer guide: Thought leader interviews from different health and development sectors

1. What does community engagement mean to you?
2. What experience has been most influential of your perspective on community engagement?
3. How has your perspective and approach to community engagement changed over time?
4. What interests you about the field of community engagement?
5. What role do you think community engagement current plays in _____________________(insert applicable health and development sector i.e. HIV/AIDS, MCH, etc.). Does it differ from other health and development sectors?
6. Generally speaking, what impact do you think community engagement has achieved in this field? What more could it achieve?
7. What areas of community engagement require more development?
8. Are there any important resources on general community engagement that you would recommend?
